# Acute and late toxicity in prostate cancer patients treated with moderately vs ultra-hypofractionated radiotherapy

**DOI:** 10.3389/fonc.2026.1843032

**Published:** 2026-05-22

**Authors:** Javier Cifuentes-Quin, Yiselle T. Garcia-Montañez, Alexandra Hurtado-Ortiz, Jhonatan Cruz, Luis Carlos Lagares, Luis A. Olarte-Licht, Maricel Licht-Ardila, Edgar Fabián Manrique-Hernández

**Affiliations:** 1 Oncology Department, Hospital Internacional de Colombia HIC - Fundación Cardiovascular de Colombia FCV - Fundación Universitaria FCV, Piedecuesta, Santander, Colombia; 2 Epidemiology Department, Hospital Internacional de Colombia HIC - Fundación Cardiovascular de Colombia FCV - Fundación Universitaria Fundación Cardiovascular de Colombia FCV, Piedecuesta, Santander, Colombia

**Keywords:** adverse effects, prostatic neoplasms, radiation dose hypofractionation, radiotherapy dosage, radiotherapy, intensity-modulated

## Abstract

**Introduction:**

Prostate cancer is the most frequently diagnosed male malignancy in Colombia. While modern radiotherapy techniques allow for hypofractionated schedules due to the low alpha/beta ratio of prostate adenocarcinoma, real-world comparative data between moderate and ultra-hypofractionation in Latin American settings remain limited.

**Objective:**

To compare acute and early late toxicity in patients with prostate cancer treated with moderately hypofractionated radiotherapy versus those treated with ultra-hypofractionated regimen.

**Methods:**

A comparative historical cohort study was conducted at a Colombian oncology center between 2018 and 2025. A total of 229 patients with non-metastatic prostate cancer (T1–T4) were enrolled (MHRT: n=143; UHRT: n=86). Toxicity was graded using CTCAE criteria. Multivariable logistic regression was used to identify independent predictors of toxicity.

**Results:**

Acute toxicity was significantly higher in the MHRT group compared to the UHRT group (25.17% vs. 11.63%; p=0.013). Similarly, late toxicity was more frequent in the MHRT group (37.06% vs. 9.30%; p<0.001), with erectile dysfunction as the most common late event. Multivariable analysis showed that UHRT was independently associated with lower odds of acute toxicity (aOR 0.44; 95% CI 0.19–0.99; p=0.048).

**Conclusion:**

UHRT showed a generally favorable safety profile, with trends toward lower acute and late toxicity compared to MHRT. In middle-income settings such as Colombia, UHRT may offer a practical approach to enhance treatment efficiency and patient access, while maintaining acceptable clinical outcomes.

## Introduction

Prostate cancer is one of the most prevalent malignancies worldwide and a leading cause of cancer-related mortality among men. According to the most recent GLOBOCAN 2022 estimates, approximately 1.5 million new cases and more than 390,000 deaths were reported globally, making it the second most frequently diagnosed cancer in men and a leading cause of cancer-related death worldwide (1). In Colombia, prostate cancer represents the most commonly diagnosed malignancy among men, with an estimated 14,460 new cases and 3,846 deaths in 2020 (2). These data underscore its significant public health burden and the need for evidence-based treatment strategies within the national context.

External beam radiotherapy (EBRT) is a cornerstone in the treatment of localized and locally advanced prostate cancer and is recommended as a definitive option in major international guidelines (3). Over the past two decades, advances such as intensity-modulated radiotherapy (IMRT) and image-guided radiotherapy (IGRT) have enhanced dose precision and target accuracy, enabled safe dose escalation while reducing exposure to adjacent organs at risk, particularly the rectum and bladder (3, 4). These developments have been associated with improved biochemical control and lower gastrointestinal (GI) and genitourinary (GU) toxicity compared with earlier three-dimensional conformal techniques.

Prostate adenocarcinoma is characterized by a low α/β ratio (approximately 1.5 Gy), indicating greater sensitivity to higher doses per fraction and providing the radiobiological rationale for hypofractionation over conventional 1.8–2.0 Gy schedules (5–7). Moderately hypofractionated radiotherapy (2.4–3.4 Gy per fraction) has become a standard of care after phase III trials such as CHHiP and PROFIT demonstrated non-inferior oncologic outcomes with comparable toxicity (8, 9). The HYPRO trial further supported its efficacy, though with slightly increased late genitourinary toxicity (10).

Ultra-hypofractionated radiotherapy (UHRT), typically delivered in five or fewer fractions using stereotactic body radiotherapy (SBRT), has emerged as an effective alternative, with phase III trials such as PACE-B demonstrating non-inferior oncologic outcomes compared with conventional or moderately hypofractionated regimens (10, 11). Although UHRT offers advantages in convenience and healthcare resource utilization, concerns persist regarding acute and late gastrointestinal (GI) and genitourinary (GU) toxicity (12).

Despite strong trial evidence, comparative real-world data on toxicity outcomes between MHRT and UHRT in Latin American high-complexity centers remain limited. The present study aims to compare acute and early late GI and GU toxicity in patients treated with moderately hypofractionated versus ultra-hypofractionated radiotherapy.

## Methods

### Study design and population

A comparative historical cohort study was conducted at a national referral oncology center in Colombia. Consecutive patients treated between May 2018 and August 2025 were included and categorized according to the radiotherapy fractionation regimen received. Patients treated with moderately hypofractionated radiotherapy (MHRT) comprised the historical cohort, whereas those treated with ultra-hypofractionated radiotherapy (UHRT/SBRT), implemented later in institutional practice, constituted the contemporary cohort (13). Treatment allocation was not randomized and was based on institutional practice evolution, clinical judgment, and technology availability over time.

Eligible participants were male patients aged ≥18 years with histopathologically confirmed prostate adenocarcinoma and non-metastatic disease (M0) at diagnosis, as determined by conventional and/or advanced imaging modalities according to institutional availability. Clinical staging was defined according to the American Joint Committee on Cancer (AJCC) TNM 8th edition classification (14). Patients with clinical stages T1–T4 were included. All patients received definitive external beam radiotherapy using IMRT/VMAT with daily IGRT. Patients were treated with either MHRT or SBRT/UHRT (14, 15). A minimum clinical follow-up of six months after completion of radiotherapy was required to ensure adequate assessment of acute and/or early late toxicity.

Exclusion criteria comprised evidence of metastatic disease (M1 or extensive nodal involvement), prior pelvic or prostate radiotherapy, treatment delivered with palliative intent, history of another pelvic malignancy previously treated with radiotherapy, insufficient follow-up (<6 months), and severe preexisting comorbidities that could preclude accurate attribution of toxicity to radiotherapy (e.g., active inflammatory bowel disease when gastrointestinal toxicity was the primary endpoint).

### Treatment protocol

All patients underwent prostate-directed definitive external beam radiotherapy delivered using highly conformal techniques, including intensity-modulated radiotherapy (IMRT) or volumetric modulated arc therapy (VMAT), with daily image-guided radiotherapy (IGRT) including positioning verification and assessment of bladder and rectal status prior to each fraction. Stereotactic body radiotherapy (SBRT) was administered exclusively in patients treated with ultra-hypofractionated regimens (UHRT). Patients in the MHRT group received 60 Gy in 20 fractions (3.0 Gy per fraction) to the prostate, corresponding to an estimated EQD2 of 77 Gy (α/β = 1.5 Gy). Patients in the UHRT group were treated with 36.25 Gy in 5 fractions (7.25 Gy per fraction) to the prostate, corresponding to an EQD2 of 90.6 Gy. When elective pelvic nodal irradiation was indicated, the pelvic lymphatic drainage received 25 Gy in 5 fractions (5 Gy per fraction), corresponding to an EQD2 of 58.3 Gy. Treatment was delivered on alternate days according to institutional workflow and patient tolerance. Dose prescription, fractionation schedules, planning approaches, and organ-at-risk constraints were established according to institutional protocols and international consensus recommendations for prostate radiotherapy, including SBRT (4, 5). All treatments were delivered following a standardized institutional protocol, all plans underwent pre-treatment quality assurance by a medical physicist, and all plans were designed by a single radiation oncologist. Fiducial markers were not used. In the absence of real-time tracking or fiducial markers, intrafraction motion was addressed indirectly through standardized bladder filling and rectal preparation protocols, daily IGRT-based positioning verification, and the application of appropriate PTV margins.

Target delineation was based on simulation computed tomography (CT). A standardized preparation protocol was applied in all patients, consisting of bladder filling using a Foley catheter with instillation of 150 cc at simulation and prior to each treatment session, along with rectal preparation including dietary measures and rectal enema to minimize rectal volume variability.

The clinical target volume (CTV) included risk-adapted volumes: low-risk patients received prostate-only CTV; intermediate-risk patients received prostate plus proximal seminal vesicles; high-risk patients without imaging evidence of seminal vesicle involvement received prostate plus proximal seminal vesicles; and high-risk patients with imaging evidence of involvement received prostate plus complete seminal vesicles. Pelvic lymph nodes were included in selected high-risk patients younger than 75 years with negative imaging and in all patients with radiologically confirmed nodal involvement (CT, MRI, or PET). For analytical purposes, CTV categories were defined according to D’Amico risk classification (low, intermediate, and high risk). CTV definition and extent were based on international guideline recommendations (ASTRO) and risk-adapted clinical decision-making. The planning target volume (PTV) was generated by applying an isotropic margin of 5 mm, except posteriorly where a reduced margin of 3 mm was used to limit rectal dose. These margins were applied uniformly across all patients.

Organs at risk included the rectum, bladder, urethra, femoral heads, penile bulb, and bowel when relevant. Treatment planning prioritized adequate target coverage while complying with institutional organ-at-risk dose constraints based on Radiation Therapy Oncology Group (RTOG)-derived protocols and international recommendations for prostate radiotherapy. These constraints were applied during plan optimization and reviewed as part of the institutional physics quality assurance process before treatment delivery.

For reproducibility, the study database included dosimetric and dose-volume variables extracted from the treatment planning system for each treatment plan, according to data availability. These included bladder and rectal dose-volume parameters, such as V40 and V60 for conventionally/moderately hypofractionated plans, and SBRT-related dose-volume metrics such as rectal V18.1Gy, V29Gy, V36cc, bladder V18.1Gy, and bladder V30cc when applicable. Additional dose distribution variables recorded in the database included maximum, minimum, and mean dose metrics. These dosimetric variables were used to characterize treatment planning across both moderately hypofractionated and ultra-hypofractionated radiotherapy groups. All treatment plans underwent institutional physics review and quality assurance prior to treatment delivery.

The indication, timing, and duration of androgen deprivation therapy (ADT) were determined based on baseline clinical risk group classification, multidisciplinary evaluation, and international guideline recommendations (NCCN and EAU guidelines). In our institutional practice, ADT was not routinely administered in low-risk patients. Patients with intermediate-risk disease received short-course ADT consisting of a single 6-month depot, whereas high-risk patients received long-term ADT consisting of leuprolide acetate administered every 6 months for a total duration of 24 months. ADT was administered according to institutional protocol based on risk group. The specific agents used included luteinizing hormone-releasing hormone (LHRH) analogs (leuprolide, goserelin, or triptorelin), with or without antiandrogens when clinically indicated.

### Study variables

Baseline clinical variables included age at diagnosis, initial prostate-specific antigen (PSA) level, clinical T stage, Gleason score/ISUP grade group, and clinical risk stratification (low, intermediate, or high risk). Baseline genitourinary function prior to radiotherapy was assessed based on clinical records, including CTCAE grade documentation and/or use of genitourinary-related medications (e.g., alpha-blockers, antimuscarinics), as a proxy for pre-existing urinary symptoms, as formal patient-reported outcome measures such as IPSS were not systematically available. Hormonal therapy (ADT) was categorized according to the specific regimen received, categories included leuprolide acetate (AL), goserelin acetate (AG), no hormonal therapy, leuprolide plus bicalutamide, leuprolide plus abiraterone, goserelin plus bicalutamide, leuprolide plus cyproterone acetate, and triptorelin. The clinical target volume (CTV) category reflected the extent of radiation fields. To improve consistency and interpretability, original treatment records were reviewed and CTV definitions were reclassified into four anatomically standardized groups: (1) prostate only, (2) prostate plus seminal vesicles (SV), (3) prostate plus pelvic lymph nodes, and (4) prostate plus SV and pelvic lymph nodes. Cases that could not be reliably categorized were grouped as “other/unclassified.” This variable enabled assessment of differences in treatment volume between fractionation regimens.

The medication category captured baseline use of urological or genitourinary-related medications, categories included alpha-blockers, antimuscarinics/autonomic nervous system (ANS) agents, GnRH analogs, flavoxate, 5-alpha reductase inhibitors, phosphodiesterase type 5 (PDE5) inhibitors, no medication, and other categories. This variable was considered a potential factor associated with treatment-related genitourinary toxicity.

Finally, relevant clinical history variables were recorded, including history of prostatic hyperplasia and prior prostate surgery.

Treatment-related variables included total prescribed dose, dose per fraction, number of fractions, planning target volume, rectal and bladder dosimetric parameters, urethral constraints, use of image guidance, and administration and duration of ADT. The primary endpoint was treatment-related toxicity. Acute toxicity was defined as adverse events occurring during radiotherapy or within 90 days after treatment completion, while early late toxicity was defined as events occurring beyond 90 days. Genitourinary (GU) and gastrointestinal (GI) toxicities were graded according to the Common Terminology Criteria for Adverse Events (CTCAE) (16), which is the current standard for toxicity reporting in oncology clinical research. The highest toxicity grade recorded during follow-up was considered for analysis.

Toxicity severity was classified according to CTCAE v5.0 criteria as grade 1 (mild symptoms not requiring intervention), grade 2 (moderate symptoms requiring medical management or limiting instrumental activities of daily living), grade 3 (severe symptoms requiring hospitalization or significantly limiting self-care activities), and grade 4 (life-threatening events). For analytical purposes, clinically significant toxicity was defined as grade ≥2.

For symptoms not explicitly defined as individual CTCAE items (e.g., stress urinary incontinence, nocturia, or mixed urinary symptoms), events were mapped to the most appropriate CTCAE category within the corresponding domain (e.g., urinary incontinence or urinary frequency), based on standardized clinical criteria. Grading was assigned according to CTCAE severity definitions, considering symptom intensity, need for medical intervention, and impact on daily activities.

Acute toxicity included genitourinary symptoms (such as actinic cystitis, stress urinary incontinence (SUI), nocturia, other and unspecified urinary symptoms) and gastrointestinal events (such as actinic proctitis). Late toxicity included erectile dysfunction (sexual domain), as well as genitourinary symptoms (e.g., dysuria or irritative urinary symptoms, urinary incontinence, weak urinary stream or obstruction, cystitis, pain, and mixed urinary symptoms) and other chronic events.

Acute toxicity types included actinic cystitis, stress urinary incontinence (SUI), nocturia, other urinary symptoms, actinic proctitis, and unspecified urinary symptoms. Late toxicity was similarly categorized and included erectile dysfunction, dysuria or irritative urinary symptoms, urinary incontinence, weak urinary stream or obstruction, cystitis, pain, mixed urinary symptoms, and other chronic events. All toxicities were categorized into genitourinary (GU), gastrointestinal (GI), and sexual domains to allow clinically meaningful interpretation of treatment-related adverse events.

### Data collection and follow-up

Consecutive patients were analyzed and toxicity outcomes were systematically collected during follow-up visits. Toxicity was assessed by treating radiation oncologists using CTCAE criteria, based on clinical evaluation complemented by patient-reported symptoms documented during routine care. All adverse events were recorded in the electronic medical record (EMR). Follow-up visits were conducted at predefined intervals according to institutional practice (typically during treatment, at 1–3 months post-treatment, and periodically thereafter), allowing standardized capture of both acute and early late toxicity. Clinical and dosimetric variables were extracted from EMR and radiotherapy planning systems.

### Statistical analysis

Descriptive statistics were used to summarize baseline characteristics. Continuous variables were reported as mean ± standard deviation or median with interquartile range, depending on distribution assessed by normality tests. Categorical variables were presented as absolute and relative frequencies. The proportion of patients experiencing acute and early late toxicity was calculated. Bivariate analyses were performed to evaluate associations between clinical or dosimetric factors and toxicity using chi-square or Fisher’s exact tests for categorical variables and Student’s t-test or Mann–Whitney U test for continuous variables, as appropriate. Multivariable logistic regression models were constructed to identify independent factors of clinically significant toxicity, including variables with clinical relevance or p < 0.20 in bivariate analysis. Statistical significance was defined as p < 0.05. Model calibration was assessed using the Hosmer–Lemeshow goodness-of-fit test, comparing observed and predicted probabilities across quantiles of estimated risk. A non-significant p-value (p>0.05) was considered indicative of adequate model fit. Analyses were conducted using Stata^®^ version 14 (StataCorp, College Station, TX).

### Ethical considerations

The study protocol was approved by the Institutional Ethics Committee (CEI 2024-07461-36). Written informed consent was obtained from all participants prior to enrollment. Data confidentiality was ensured throughout the study.

## Results

A total of 229 patients were included (MHRT: n=143; UHRT: n=86). Age, Gleason score distribution, NCCN risk category, and clinical T stage were comparable between groups (all p>0.05). Baseline PSA levels and follow-up PSA values did not differ significantly. Hormonal therapy patterns and CTV definition varied between groups (p=0.005 and p<0.001, respectively). Other variables, including history of prostatic hyperplasia and prior surgery, were similarly distributed. Median follow-up time for the overall cohort was 26.8 months (IQR: 12.3–61.5), with significantly longer follow-up in the moderately hypofractionated radiotherapy group compared with the ultra-hypofractionated group (62.5 [56.4–75.0] vs. 12.1 [7.0–17.8] months, respectively; p<0.001) (**Table 1**).

**Table 1:** Baseline demographic, tumor, and treatment characteristics according to hypofractionation regimen (moderate vs ultra-hypofractionated radiotherapy).

Characteristic	Overall (n=229)	MHRT (n=143)	UHRT (n=86)	P-value
Age, years (median, P25–P75)	72 (67–77)	72 (67–77)	72.5 (67–77)	0.981
Initial PSA (median, IQR)	14.05 (8.75–27.56)	14.70 (9.39–31.5)	14.00 (6.44–23.77)	0.109
Follow-up PSA (median, IQR)	0.06 (0.01–0.26)	0.06 (0.01–0.23)	0.07 (0.01–0.30)	0.184
Gleason score, n (%)				0.445
6 (3 + 3)	43 (18.78)	30 (20.98)	13 (15.12)	
7 (3 + 4)	36 (15.72)	17 (11.89)	19 (22.09)	
7 (4 + 3)	54 (23.58)	34 (23.78)	20 (23.26)	
8 (4 + 4)	68 (29.70)	45 (31.47)	23 (26.74)	
9 (4 + 5)	18 (7.86)	11 (7.69)	7 (8.14)	
9 (5 + 4)	6 (2.62)	3 (2.10)	3 (3.49)	
10 (5 + 5)	3 (1.31)	2 (1.40)	1 (1.16)	
Clinical	1 (0.44)	1 (0.70)	0 (0.00)	
Risk classification (NCCN), n (%)				0.844
High	157 (69.16)	98 (68.53)	59 (70.24)	
Intermediate	59 (25.99)	37 (25.87)	22 (26.19)	
Low	10 (4.41)	7 (4.90)	3 (3.57)	
Very high	1 (0.44)	1 (0.70)	0 (0.00)	
Clinical T stage group, n (%)				0.976
Missing	4 (1.75)	3 (2.10)	1 (1.16)	
T1	47 (20.52)	28 (19.58)	19 (22.09)	
T2	104 (45.41)	65 (45.45)	39 (45.35)	
T3	60 (26.20)	38 (26.57)	22 (25.58)	
T4	14 (6.11)	9 (6.29)	5 (5.81)	
Hormonal therapy, n (%)				0.024
No ADT	8 (3.49)	4 (2.80)	4 (4.65)	
LHRH agonist ± antiandrogen	217 (94.76)	139 (97.20)	78 (90.70)	
LHRH agonist + ARPI (abiraterone)	4 (1.75)	0 (0.00)	4 (4.65)	
CTV category, n (%)				<0.001
Prostate	31 (13.54)	6 (4.20)	25 (29.07)	
Prostate + seminal vesicles	135 (58.95)	127 (88.81)	8 (9.30)	
Prostate + pelvic nodes	39 (17.03)	0 (0.00)	39 (45.35)	
Prostate + SV + pelvic nodes	18 (7.86)	4 (2.80)	14 (16.28)	
Unclassified	6 (2.62)	6 (4.20)	0 (0.00)	
Medication category, n (%)				<0.001
Alpha-blockers	27 (11.79)	27 (18.88)	0 (0.00)	
Antimuscarinics/ANS agents	7 (3.06)	7 (4.90)	0 (0.00)	
GnRH analogs	6 (2.62)	6 (4.20)	0 (0.00)	
Flavoxate	4 (1.75)	0 (0.00)	4 (4.65)	
5-alpha reductase inhibitors	1 (0.44)	1 (0.70)	0 (0.00)	
PDE5 inhibitors	5 (2.18)	5 (3.50)	0 (0.00)	
No	157 (68.56)	87 (60.84)	70 (81.40)	
Other categories	28 (12.23)	10 (6.99)	18 (20.93)	
History of prostatic hyperplasia, n (%)	28 (24.78)	4 (14.81)	24 (27.91)	0.169
Prior surgery n (%)	10 (4.37)	5 (3.50)	5 (5.81)	0.406
Follow-up time, m median (IQR)	26.8 (12.3-61.5)	62.5 (56.4–75.0)	12.1 (7.0-17.8)	< 0.001

MHRT, Moderate Hypofractionated Radiotherapy; UHRT, Ultra-Hypofractionated Radiotherapy; PSA, prostate-specific antigen; m, months; NCCN, National Comprehensive Cancer Network; CTV, clinical target volume; SV, seminal vesicles; GnRH, gonadotropin-releasing hormone; PDE5, phosphodiesterase type 5; ANS, autonomic nervous system; ADT, Androgen Deprivation Therapy; LHRH, luteinizing hormone–releasing hormone; ARPI, androgen receptor pathway inhibitor (abiraterone). Continuous variables were compared using the Mann–Whitney U test, and categorical variables using the chi-square test. Initial PSA values were available for 218 patients (moderate group n=139; ultra group n=79), and follow-up PSA values for 219 patients (moderate group n=141; ultra group n=78). PSA: ng/mL.

Acute toxicity occurred in 20.09% of patients and was more frequent in the moderate hypofractionation group compared with the ultra-hypofractionation group (25.17% vs. 11.63%, p = 0.013). When stratified by toxicity domain, most acute events were genitourinary (GU), followed by gastrointestinal (GI) toxicity. No significant differences were observed in the distribution of specific acute toxicity types between groups (p = 0.096). Early late toxicity was observed in 27.51% of patients and was higher in the moderate group (37.06% vs. 9.30%, p < 0.001). By domain, genitourinary toxicity remained the most frequent, while sexual toxicity—primarily erectile dysfunction—was the most common individual late event and was significantly more frequent in the moderate group (13.29% vs. 1.16%, p < 0.001) (**Table 2**).

**Table 2:** Acute and late toxicity by radiotherapy fractionation regimen.

Variable	Overall n=229 (%)	Moderate n=143 (%)	Ultra n=86 (%)	P-value
ACUTE TOXICITY				
With toxicity	46 (20.09)	36 (25.17)	10 (11.63)	0.013
Without toxicity	183 (79.91)	107 (74.83)	76 (88.37)	
Type of acute toxicity*				
Actinic cystitis	18 (7.86)	15 (10.49)	3 (3.49)	0.096
SUI	6 (2.62)	6 (4.20)	0 (0.00)	
Nocturia	4 (1.75)	4 (2.80)	0 (0.00)	
Other urinary symptoms	11 (4.80)	7 (4.90)	4 (4.65)	
Actinic proctitis	5 (2.18)	3 (2.10)	2 (2.33)	
Urinary symptoms (unspecified)	2 (0.87)	1 (0.70)	1 (1.16)	
EARLY LATE TOXICITY				
With toxicity	63 (27.51)	53 (37.06)	8 (9.30)	<0.001
Without toxicity	166 (72.49)	90 (62.94)	78 (90.70)	
Type of chronic symptoms*				
Erectile dysfunction	20 (8.73)	19 (13.29)	1 (1.16)	<0.001
Dysuria/irritative symptoms	13 (5.68)	11 (7.69)	2 (2.33)	
Urinary incontinence	5 (2.18)	5 (3.50)	0 (0.00)	
Weak stream/obstruction	4 (1.75)	3 (2.10)	1 (1.16)	
Cystitis	2 (0.87)	0 (0.00)	2 (2.33)	
Pain	2 (0.87)	2 (1.40)	0 (0.00)	
Mixed symptoms	6 (2.62)	6 (4.20)	0 (0.00)	
Other	9 (3.93)	7 (4.90)	2 (2.33)	

Data are presented as numbers (percentage). Acute and chronic toxicity were categorized as present or absent. Specific toxicity types are shown descriptively and are not mutually exclusive. Comparisons between moderately hypofractionated and ultra-hypofractionated regimens were performed using the chi-square test or Fisher’s exact test, as appropriate. A p-value <0.05 was considered statistically significant. SUI, stress urinary incontinence.

**Figure 1:**
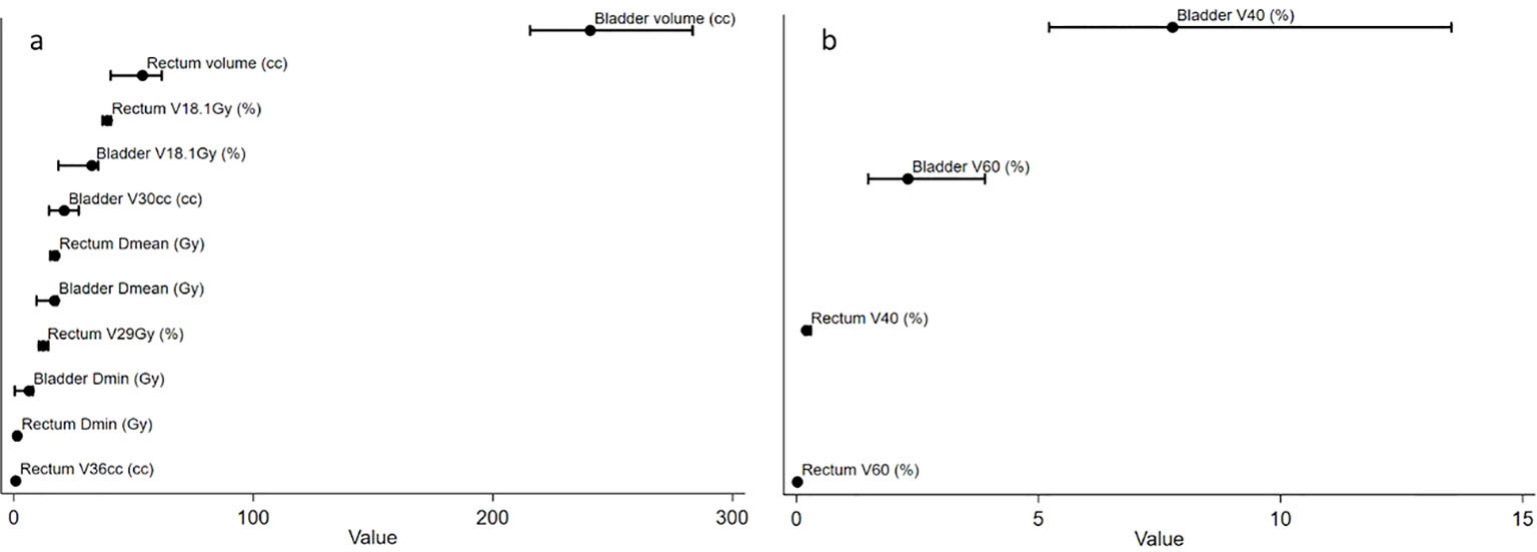
Descriptive dosimetric parameters for rectum and bladder (organs at risk). **(A)** Ultra-hypofractionation group. **(B)** Moderately hypofractionated group. Points represent median values and horizontal bars indicate interquartile ranges (IQR).

For the rectum, the median volume was 53.7 cc (IQR: 40.5–61.7), with a median mean dose (Dmean) of 17.2 Gy (IQR: 15.2–18.2). The median V18.1Gy was 39.1%, while high-dose volumes were low (V40: 0.21%; V60: 0.02%). For the bladder, the median volume was 240.6 cc (IQR: 215.5–283.4), with a median Dmean of 17.0 Gy (IQR: 9.5–18.2). The median V18.1Gy was 32.6%, and V30cc was 21.1 cc. In the extended analysis (n=140–142), median bladder V40 and V60 were 7.8% and 2.3%, respectively (**Figure 1**).

In the multivariable analysis (**Table 3**), ultra-hypofractionation was independently associated with lower odds of acute toxicity compared with moderate hypofractionation (adjusted OR 0.44; 95% CI 0.19–0.99; p = 0.048). Conversely, baseline medication use was associated with increased odds of acute toxicity (adjusted OR 3.09; 95% CI 1.54–6.19; p = 0.001). The clinical risk group was not associated with acute toxicity. The Hosmer–Lemeshow goodness-of-fit test showed no evidence of lack of fit (χ²=8.56; p=0.38), indicating adequate calibration of the logistic regression model for acute toxicity.

**Table 3:** Multivariable logistic regression analysis of factors associated with acute toxicity.

Variable	Adjusted OR	95% CI	P-value
Fractionation regimen			
Ultra vs Moderate	0.44	0.19 – 0.99	0.048
Risk group			
Intermediate vs Low	0.66	0.11 – 3.97	0.649
High vs Low	1.22	0.23 – 6.49	0.816
Very high	—	—	—
Medication use			
Any medication vs None	3.09	1.54 – 6.19	0.001

Data are presented as adjusted odds ratios (OR) with 95% confidence intervals (CI). The moderate fractionation regimen and low-risk group were used as reference categories.

In the multivariable analysis (**Table 4**), higher NCCN risk category was associated with increased odds of late toxicity; however, effect estimates were imprecise, with wide confidence intervals, likely reflecting the limited number of events in the reference group. Baseline medication use (adjusted OR 4.63; p < 0.001) and prior surgery (adjusted OR 9.14; p = 0.018) were also independently associated with late toxicity. The Hosmer–Lemeshow goodness-of-fit test indicated no evidence of poor model fit for late toxicity (χ²=2.41; p=0.661). This suggests adequate agreement between observed and predicted probabilities across risk groups, supporting good model calibration.

**Table 4:** Multivariable logistic regression analysis of factors associated with late toxicity.

Variable	Adjusted OR	95% CI	P-value
NCCN risk group			
Intermediate vs Low	67.13	2.40 – 1881.52	0.013
High vs Low	69.25	2.57 – 1864.76	0.012
Very high	—	—	—
Medication use (Yes vs No)	4.63	2.09-10.29	<0.001
Surgery (Yes vs No)	9.14	1.46 – 57.02	0.018

Data are presented as adjusted odds ratios (OR) with 95% confidence intervals (CI). The moderate fractionation regimen and low-risk group were used as reference categories.

## Discussion

In this cohort conducted at a high-complexity referral center in Colombia, UHRT demonstrated a favorable toxicity profile compared with MHRT, without evidence of clinically meaningful excess acute or early late adverse events. Erectile dysfunction was the most frequently observed late event overall. Importantly, toxicity patterns in the UHRT group were consistent with contemporary international standards for image-guided intensity-modulated radiotherapy.

Our findings are aligned with results from randomized trials evaluating ultra-hypofractionation in localized prostate cancer. The PACE-B trial demonstrated that stereotactic body radiotherapy (SBRT) achieved comparable safety outcomes to conventionally fractionated regimens, with no clinically significant increase in acute or late genitourinary or gastrointestinal toxicity during follow-up (11). Similarly, long-term results from the HYPO-RT-PC confirmed non-inferiority of ultra-hypofractionation, with sustained disease control and acceptable early late toxicity profiles (11, 17). These data support the biological rationale of prostate cancer’s low α/β ratio and reinforce the safety of larger fraction sizes when delivered with modern techniques.

In contrast, moderate hypofractionation has been validated through large multicenter trials such as CHHiP, which established its non-inferiority to conventional fractionation and confirmed acceptable long-term toxicity (18). However, differences in treatment volumes, patient selection, and use of androgen deprivation therapy may influence toxicity patterns across regimens. In real-world settings, these clinical variables often guide treatment allocation and may partially explain observed differences between fractionation schedules. Additionally, although ADT use differed between groups, its indication followed standardized risk-adapted institutional criteria aligned with international guidelines, which may mitigate potential confounding by indication.

In the adjusted analysis, ultra-hypofractionation was independently associated with a lower likelihood of acute toxicity, whereas baseline medication use emerged as a possible predictor of acute events. These findings are consistent with recent secondary analyses of contemporary SBRT trials and real-world cohorts showing that baseline urinary symptoms and pre-treatment genitourinary medication use are significant predictors of acute urinary morbidity (19, 20). Similarly, early late toxicity in our cohort was independently associated with higher NCCN risk category, prior surgery, medication use, underscoring the multifactorial nature of late treatment-related adverse events. Recent studies have highlighted that patient-related clinical vulnerability and baseline functional status may have a greater impact on early late toxicity than fractionation regimen alone (21, 22).

From a technical standpoint, strict adherence to contemporary organ-at-risk constraints and the use of daily image guidance likely contributed to the favorable safety outcomes observed in the UHRT group. However, the more favorable toxicity profile observed with UHRT may also be partially explained by differences in rectal and bladder dosimetric exposure between treatment regimens and therefore should not be attributed solely to the fractionation schedule itself. Current consensus recommendations emphasize minimizing intermediate- and high-dose exposure to rectum and bladder to reduce late genitourinary and gastrointestinal morbidity (23). When such constraints are respected, SBRT has demonstrated reproducible safety across institutions.

The relevance of our findings is particularly notable in the context of a middle-income country. Colombia faces persistent geographic and socioeconomic disparities in access to specialized oncologic care (24). Our institution receives patients from rural and underserved regions, where prolonged treatment schedules may impose substantial indirect costs and logistical burdens. Shortening radiotherapy duration through UHRT reduces the number of hospital visits, improves treatment adherence, and decreases the likelihood of interruptions. Additionally, hypofractionation strategies optimize machine time and increase radiotherapy capacity, an important consideration in resource-constrained environments. Global oncology analyses have highlighted hypofractionation as a key approach to expanding equitable access to radiotherapy services in low- and middle-income countries (25).

Ultra-hypofractionation can be safely implemented outside strictly controlled trial settings when appropriate technology, planning standards, and quality assurance measures are in place. Multi-institutional experiences and pooled analyses have demonstrated low rates of severe toxicity and favorable biochemical control with stereotactic body radiotherapy (SBRT) delivered in routine clinical practice (26, 27). Furthermore, professional society guidelines have established technical standards and organ-at-risk constraints that support the safe implementation of hypofractionated regimens when modern image-guided techniques are used (23). These findings support the broader adoption of UHRT in carefully selected patients, particularly in high-demand centers seeking to balance clinical effectiveness, safety, and healthcare system sustainability.

### Limitations

The observed differences between groups should be interpreted in the context of a non-randomized design, as treatment allocation was influenced by the temporal implementation of SBRT and clinical decision-making, introducing potential selection bias, an inherent limitation of observational comparative studies (23, 28, 29). Additionally, differences in baseline characteristics, dosimetric parameters, and organ-at-risk constraints between MHRT and UHRT may have contributed to the observed toxicity patterns, and residual confounding related to treatment planning cannot be excluded. Follow-up duration differed significantly between groups due to the sequential implementation of ultra-hypofractionated radiotherapy within institutional practice. Therefore, late toxicity outcomes in the UHRT cohort, particularly erectile dysfunction, should be interpreted with caution, as the shorter median follow-up duration may underestimate the true incidence of delayed adverse events that typically develop over longer observation periods.

Toxicity assessment was based on clinician-reported outcomes using CTCAE criteria rather than validated patient-reported outcome measures (PROMs), which may underestimate subjective symptoms such as urinary or sexual dysfunction and represents a potential source of measurement bias. Future studies incorporating PROMs are warranted to provide a more comprehensive assessment of treatment-related toxicity.

Furthermore, longer follow-up is required to fully characterize early late toxicity and long-term oncologic outcomes, as demonstrated in extended analyses of randomized hypofractionation trials (30). Finally, the association observed between the NCCN risk group and early late toxicity should be interpreted with caution. The large effect estimates and wide confidence intervals likely reflect sparse data and a limited number of events in the reference category, which may result in model instability and overestimation of effect size. Therefore, these findings should be considered indicative of the direction of association rather than precise estimates.

The higher frequency of erectile dysfunction observed in the moderately hypofractionated group compared with the SBRT group should be interpreted with caution, as the magnitude of this difference exceeds that reported in randomized trials such as PACE-B (11), where SBRT demonstrated a comparable toxicity profile. This finding may reflect residual confounding or differences in baseline characteristics and outcome assessment inherent to the study design and should therefore be considered exploratory.

## Conclusion

Ultra-hypofractionated radiotherapy appears to be a safe and pragmatic strategy for the management of localized prostate cancer in a middle-income setting. Beyond its radiobiological advantages, UHRT represents an opportunity to improve access, reduce socioeconomic barriers, and enhance radiotherapy efficiency without compromising patient safety. However, these findings should be interpreted in the context of potential differences in treatment planning and dosimetric parameters between regimens. The expansion of hypofractionation may contribute to optimizing radiotherapy capacity and partially addressing infrastructure constraints in low- and middle-income settings.

## Data Availability

The raw data supporting the conclusions of this article will be made available by the authors, without undue reservation.
